# First experience with third‐party validations: A robust calibration and QA procedure for proton FLASH delivery

**DOI:** 10.1002/acm2.70269

**Published:** 2025-09-27

**Authors:** Chih‐Chiang Chang, Balaji Selvaraj, Xingyi Zhao, Jacob Rembish, Paige A. Taylor, Alexander Bookbinder, Chingyun Cheng, J. Isabelle Choi, Charles B. Simone, Haibo Lin, Minglei Kang

**Affiliations:** ^1^ New York Proton Center New York New York USA; ^2^ The University of Texas MD Anderson Cancer Center Houston Texas USA; ^3^ Department of Human Oncology University of Wisconsin‐Madison Madison Wisconsin USA

**Keywords:** calibration, credentialing, FLASH radiotherapy, pencil beam scanning, quality assurance (QA)

## Abstract

**Background:**

Proton FLASH radiotherapy, delivering ultra‐high dose rates, shows promise in reducing normal tissue toxicity while maintaining tumor control. However, accurate dosimetry and quality assurance (QA) for FLASH remain challenging due to the extreme dose rates involved. Developing reliable calibration and QA procedures is crucial for advancing FLASH towards clinical implementation.

**Purpose:**

To present an effective routine calibration and QA procedure for proton FLASH delivery to ensure high‐quality dosimetry performance for preclinical and clinical delivery.

**Methods:**

A high temporospatial resolution strip ionization chamber array (SICA) detector was mounted to the treatment nozzle, which was calibrated using an Advanced Markus ion chamber (IC) under variable dose and ultra‐high dose rates. The calibration curve was used to monitor the delivery in real‐time. The maximum beam current was 215 nA at the isocenter at the highest energy of the cyclotron (250 MeV). Third‐party IROC (Imaging and Radiation Oncology Core) thermoluminescent dosimeters (TLDs) were requested to verify the accuracy and effectiveness of the calibration and validation procedure. A transmission field with a field size of 3 × 3 cm^2^ was designed to deliver 2000 cGy doses with a field‐averaged dose rate varying from 36 to 76.5 Gy/s.

**Results:**

The entire calibration process took less than 10 minutes as part of routine daily QA. The calibration curve between the SICA detector and IC demonstrated an *R*‐value of almost 1.00. The SICA detector measured each delivery, providing critical data for FLASH analysis, including field size, dose, delivery time, and dose rate. Based on real‐time dose and dose rate monitoring, the planned and delivered doses were within 1% accuracy according to third‐party TLD measurements.

**Conclusion:**

As demonstrated by IROC measurements, effective calibration and QA using a SICA detector for proton FLASH delivery can ensure high accuracy in real‐time dose and dose rate monitoring, paving the way for future clinical applications.

## 1 INTRODUCTION

FLASH radiotherapy (FLASH‐RT) has emerged as a promising advance in radiation therapy, delivering ultra‐high dose rates (UHDRs) typically greater than 40 Gy/s.^1^, ^2^, ^3^ This technique has been shown to significantly reduce normal tissue toxicity while maintaining comparable tumor control, a phenomenon known as the FLASH effect.^4^, ^5^ Preclinical studies have extensively documented these benefits, highlighting the potential of FLASH‐RT to widen the therapeutic window.^6^, ^7^, ^8^

While most preclinical data reporting FLASH effects have been conducted with electron radiation, the clinical translation of FLASH‐RT using electrons is inherently limited in treating deep‐seated tumors and large target volumes. Electron‐based FLASH‐RT is limited by the physical properties of electron interactions, including energy loss through Coulomb interactions with orbital electrons and angular deflection through Coulomb interactions with nuclei, which together restrict its penetration depth and efficacy for deep‐seated targets.^9^, ^10^ This has led researchers to explore alternative modalities, with proton therapy emerging as a promising candidate due to its superior dose conformity and capability to treat deep‐seated targets effectively. In fact, the first prospective clinical trial investigating the FLASH effect was recently conducted using proton therapy.^11^, ^12^ Proton pencil beam scanning (PBS) using Bragg peaks, in particular, offers advanced dosimetric advantages that make it suitable for the clinical translation of FLASH‐RT.^13^, ^14^, ^15^, ^16^

Accurate dosimetry in proton PBS FLASH‐RT is critical due to the unique spatiotemporal correlation of the delivered radiation.^6^, ^13^, ^14^ This correlation complicates the definition and characterization of dose rate, especially since FLASH‐RT with proton PBS requires delivering large beam currents over a short delivery time.^17^, ^18^, ^19^ Current technologies often lack the necessary spatial and temporal resolution to monitor these parameters accurately.^13^, ^14^ For instance, in the context of FLASH‐RT with proton PBS, current methods, such as the use of traditional ion chamber (IC) arrays, point dosimeters, log file analysis, and radiochromic films, present limitations in either spatial resolutions or lack of real‐time feedback capabilities, making them less suitable for preclinical or clinical applications.^13^, ^14^ Furthermore, the definition of voxel‐wise dose rate in proton beam therapy, particularly for PBS techniques, has not reached a consensus, prompting important conceptual questions and necessitating the collection of 2D dose delivery data. PBS delivery differs fundamentally from traditional double‐scattered (DS) proton beams. While DS systems deliver dose uniformly across the entire field, PBS utilizes a narrow beam that systematically scans the target volume, enabling both intensity modulation of individual beamlets and superior dose conformality.^20^, ^21^ Early investigations have utilized field‐averaged dose rate (FADR), defined as the total field dose divided by delivery time. However, this metric may inadequately represent localized dose rates, especially in larger treatment volumes.^22^ Besides FADR, we also used the local average dose rate (LADR)^22^ in evaluating the FLASH ratio (V_40Gy/s_). This more granular, voxel‐specific metric offers a refined approach to quantifying the temporal aspects of dose delivery in PBS proton therapy.^15^, ^23^

Addressing these challenges, a novel two‐dimensional (2D) parallel‐plate strip ionization chamber array (SICA) has been introduced.^6^, ^13^, ^14^ The SICA offers high spatiotemporal resolution, making it well‐suited for monitoring beam spot positions, profiles, and dose rates in proton PBS FLASH‐RT. A recent study characterized the SICA and demonstrated its application in proton PBS delivery systems, highlighting its potential for both clinical and ultrahigh dose rate settings.^13^, ^24^ Additionally, the commissioning of a 250 MeV research beamline for proton FLASH‐RT has been detailed, demonstrating its potential applications in preclinical studies.^14^ This dedicated research beamline aims to advance the understanding and implementation of proton FLASH‐RT, providing a crucial platform for further translational research efforts.

This technical note presents an effective routine calibration and quality assurance (QA) procedure for proton FLASH delivery, designed to ensure high‐quality dosimetry performance in both preclinical and clinical settings. The accuracy and effectiveness of the proposed procedure have been validated through third‐party validation, further demonstrating its reliability and robustness. These advancements underscore the critical role of innovative detector technologies and dedicated research infrastructures in the successful clinical translation of proton FLASH‐RT, paving the way for more effective and safer cancer treatments.

## 2 METHOD

### 2.1 Experimental setup

**FIGURE 1:**
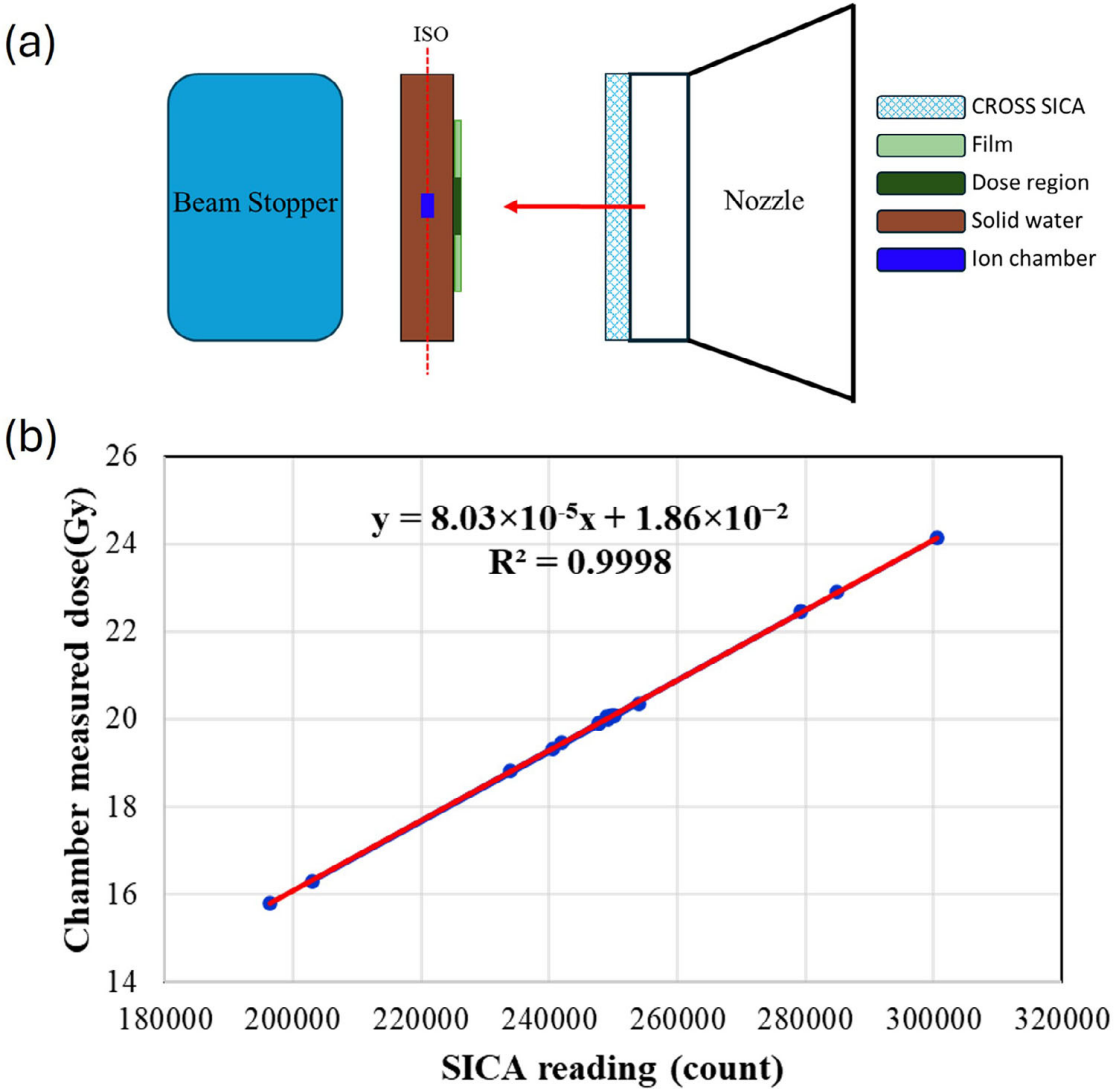
(a) The SICA was mounted onto the nozzle and calibrated using an Advanced Markus ion chamber. An EBT‐XD film was used to ensure the chamber was positioned at the field center; (b) the daily calibration curve was obtained with a proton transmission beam of 250MeV under beam currents from 100 to 215 nA.

The FLASH delivery mode utilized 250 MeV proton beams, the highest energy of our ProBeam system (Varian Medical Systems, Palo Alto, CA, USA), which has been previously commissioned for proton FLASH animal irradiation studies.^6^, ^13^, ^14^ The experimental setup was designed to ensure accurate calibration and analysis of the proton beam's characteristics. For the calibration setup (Figure 1), the proton beam was delivered through a sequence of components to guarantee precise measurements and comprehensive analysis of the beam properties. The beam first traversed the SICA detector (Liverage Biomedical Inc., Hsinchu, Taiwan), which offers a high spatiotemporal resolution of acquisition frequency of 20 kHz and 2 mm strip pitch.^13^ The high‐resolution capabilities of the SICA allowed for detailed tracking and recording of the proton beam's path and intensity, providing data for subsequent analysis. An EBT‐XD film was attached to the SICA detector to cross‐check the dose profile. The EBT‐XD film has a dynamic range from 0.4 to 40 Gy, with sensitivity and accuracy, making it an ideal choice for this purpose.^25^ Next, the beam encountered the Advanced Markus chamber (PTW, Freiburg, Germany), an ionization chamber that has demonstrated accuracy in measuring PBS FLASH dosimetry.^26^, ^27^ The Advanced Markus chamber was placed at the isocenter, with a 2 cm buildup to measure the absolute dose, which was used to calibrate the SICA detector. Lastly, for all measurements, including dose calibration, validation, and dose rate quantification, we utilized consistent beam parameters. A transmission field with dimensions of 3 × 3 cm^2^ was implemented across all tests. The field was designed to deliver 2000 cGy doses throughout the experiments. 5 mm spot spacing was maintained at uniform intervals within this field, and the FADR was systematically varied from 36 to 76.5 Gy/s, corresponding to beam currents ranging from 100 to 215 nA. This range of operating conditions allowed us to evaluate dose delivery performance across clinically relevant beam intensities.

### 2.2 Dose calibration and validation

The SICA detector was positioned at the beam path during FLASH irradiation to measure real‐time dose and dose rate using proprietary software developed in‐house. The detector's operating principle involves collecting ionization‐induced charges at individual electrodes, amplifying these signals, and converting them to digital format through analog‐to‐digital processing to produce SICA readings (Figure 1b). The SICA system captures one‐dimensional dosimetric profiles in both *X* and *Y* directions using an array of 127 x‐strips and 127 y‐strips where each strip functions as an independent ionization chamber channel. Our data acquisition system combines fast analog processing with high‐frequency digital sampling, achieving a measurement frequency of 20 kHz (50 µs intervals). The DAQ system continuously records signals from all 254 channels (127 x‐strips + 127 y‐strips) throughout the entire irradiation period, storing both the instantaneous readings and their temporal sequence.

These one‐dimensional signal profiles serve as input for a convolution superposition algorithm that reconstructs two‐dimensional dose distributions.^13^, ^28^ The reconstruction process involves several key steps. First, we analyze the spatial and temporal data from each SICA measurement to extract delivery duration and beam spot positions in both *X* and *Y* directions. Next, our software fits double‐Gaussian functions to the *X* and *Y* profile data separately. This mathematical modeling enables us to reconstruct the complete two‐dimensional dose distribution at each temporal sampling point. The cumulative dose is then calculated by summing the contributions from all delivered beam spots across the entire treatment duration.

The final step combines the reconstructed dose maps with their corresponding temporal information to generate spatial dose rate distributions across the beam profile. To ensure absolute dose accuracy, we used measurements from an Advanced Markus chamber to calibrate the SICA detector signals to isocenter dose values at various dose and dose rate levels (Figure 1b).

### 2.3 Dose rate quantification

For dose rate analysis, we examined both FADR and LADR. FADR was calculated by dividing the central axis dose by the total time used to deliver the entire field, representing the most conservative measurement method.^14^ In addition, LADR was defined below on a voxel‐wise basis as previously described.^6^, ^14^, ^23^

$$D_{LADR}\;\left(\vec{x}\right)=\frac{D\left(\vec{x}\right)-2d^{\prime }}{t_{1}-t_{0}}$$

$D(\vec{x})$ represents the total cumulative dose delivered to position *x* throughout the irradiation process. The LADR function $D_{LADR}\left(\vec{x}\right)$ at position *x* is calculated using several key parameters. The parameter d' serves as a low‐dose cutoff threshold selected. Two critical time points are identified:$\;t_{0}$, which marks the moment when position x initially receives the threshold dose d', and $\;t_{1}$, which indicates the time when position x receives a dose equal to $D\left(\vec{x}\right)-d^{\prime }$. The difference between these time points $\;t_{1}-t_{0}$ forms the denominator in the equation, while the numerator consists of $D\left(\vec{x}\right)-2d^{\prime }$, representing the effective dose change over this time interval.

PBS delivers radiation as a series of individually targeted beam spots, in contrast to scattering‐based techniques that irradiate the entire field uniformly and simultaneously. This sequential delivery adds complexity to dose rate assessment. The LADR metric accounts for the detailed time structure of beam delivery—including individual spot times, scanning intervals, and the cumulative dose and duration received by each region of interest. Compared to other definitions,^14^, ^22^, ^23^ LADR adopts a more conservative approach, offering a voxel‐level measure for evaluating dose rates.

The log files contained time‐stamped records of various machine parameters, including spot beam‐on time, spot position, and MUs delivered. An in‐house analytical program was developed to reconstruct the dose rate using these log file records. The program extracted the delivered spot positions, spot weights, and delivery timing sequence to generate a four‐dimensional dose rate matrix. This reconstruction approach accounts for the temporal and spatial variations in dose delivery, enabling a comprehensive characterization of the treatment process. The reconstructed dose rate distributions were subsequently compared with the SICA measured dose rate distributions to evaluate the accuracy of the delivery system and to verify the consistency between planned and delivered treatments.

### 2.4 IROC validation

To validate our calibration and QA performance, we requested Image and Radiation Oncology Core (IROC) thermoluminescent dosimeters (TLDs) for dose validation.^29^, ^30^ The three groups of TLDs were embedded in a miniature acrylic block, and a total of 9 blocks were shipped to our center. Each IROC‐provided acrylic block contained TLDs positioned according to IROC's standardized specifications for proton beam validation. The blocks were handled following IROC's guidelines to maintain dosimetric integrity. The 8 blocks were utilized for measuring dose rates. The remaining 1 block was discarded due to a beam interruption. Each block was centrally positioned within the irradiation field on the treatment table. Four distinct FADR dose rates were selected to represent a range of clinical scenarios: 36, 53, 70, and 76.5 Gy/s, corresponding to beam currents of 100, 150, 200, and 215 nA, respectively. For each FADR, two blocks were used to repeat the delivery, resulting in six readings per dose rate. The water‐equivalent depth of the blocks is 1.8 cm, with 0.2 cm of additional water‐equivalent phantom material used to align the output measurement point with the TLD depth, ensuring dosimetric accuracy. The additional water‐equivalent material was carefully positioned to maintain the precise measurement depth required by the IROC protocol. The block positioning was verified to ensure accurate placement within the 3 × 3 cm^2^ field. The proton beam was delivered with a field size of 3 × 3 cm^2^ with 5 mm spot spacing, and the TLDs were exposed to a total dose of 2000 cGy at the specified dose rates. Comprehensive documentation of the irradiation parameters, including calibration procedures, field dimensions, measurement depth, and supplementary buildup material, was recorded on the TLD irradiation forms. Following irradiation, the TLDs were returned to IROC for analysis in accordance with their protocols to facilitate reliable and accurate third‐party credentialing for the proton beam output. This rigorous approach to calibration and dosimetry ensured that all experimental data were accurate, reproducible, and suitable for further analysis in the context of proton PBS FLASH‐RT and future clinical trials.

## 3 RESULT

### 3.1 Dose calibration and validation

Validation measurements of the calibrated dose measured by the SICA at a proton beam current of 215 nA using the one‐shot setup are presented in Figure 1a. The doses measured by the SICA exhibited excellent linearity with the measurements by the Advanced Markus Chamber. This close correspondence indicates the high accuracy of the SICA in UHDR conditions. Furthermore, a strong correlation between the SICA and IC measurements was evidenced by the high linearity (*R* ^2^ = 0.9998).

**FIGURE 2:**
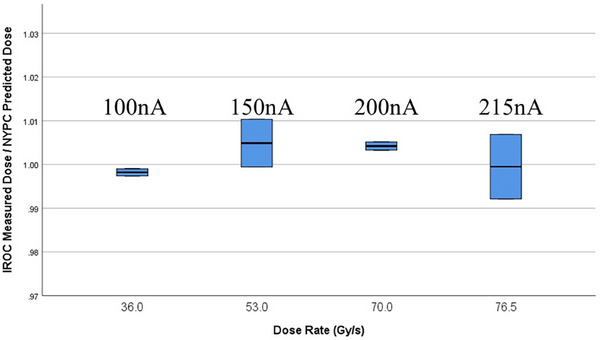
The planned and IROC TLD measured dose comparison under 4 different FADR dose rates: 36, 53, 70, and 76.5 Gy/s. Each dose rate used two blocks with a total of 6 readings to confirm the delivered dose.

To quantify the precision of dose delivery, the ratio of IROC TLD measured doses to the planned doses for each of the four Field Average Dose Rate (FADR) values was calculated (Figure 2), which directly corresponds to the beam current settings used in our experimental design. Those findings revealed a remarkable consistency across all measurements. Specifically, the ratios for all four dose rates were found to be very close to 1, with values ranging from 0.992 to 1.010. All four measurements exhibited standard deviations less than 0.01, with values ranging from 0.001 to 0.010. This proximity to unity is a strong indicator of the high degree of accuracy achieved in our dose delivery system, demonstrating that the actual delivered doses align exceptionally well with the planned doses across the entire range of tested dose rates. These results collectively suggest that our radiation delivery system maintains excellent accuracy and precision across a wide range of clinically relevant dose rates.

### 3.2 Dose distribution of SICA and film

**FIGURE 3:**
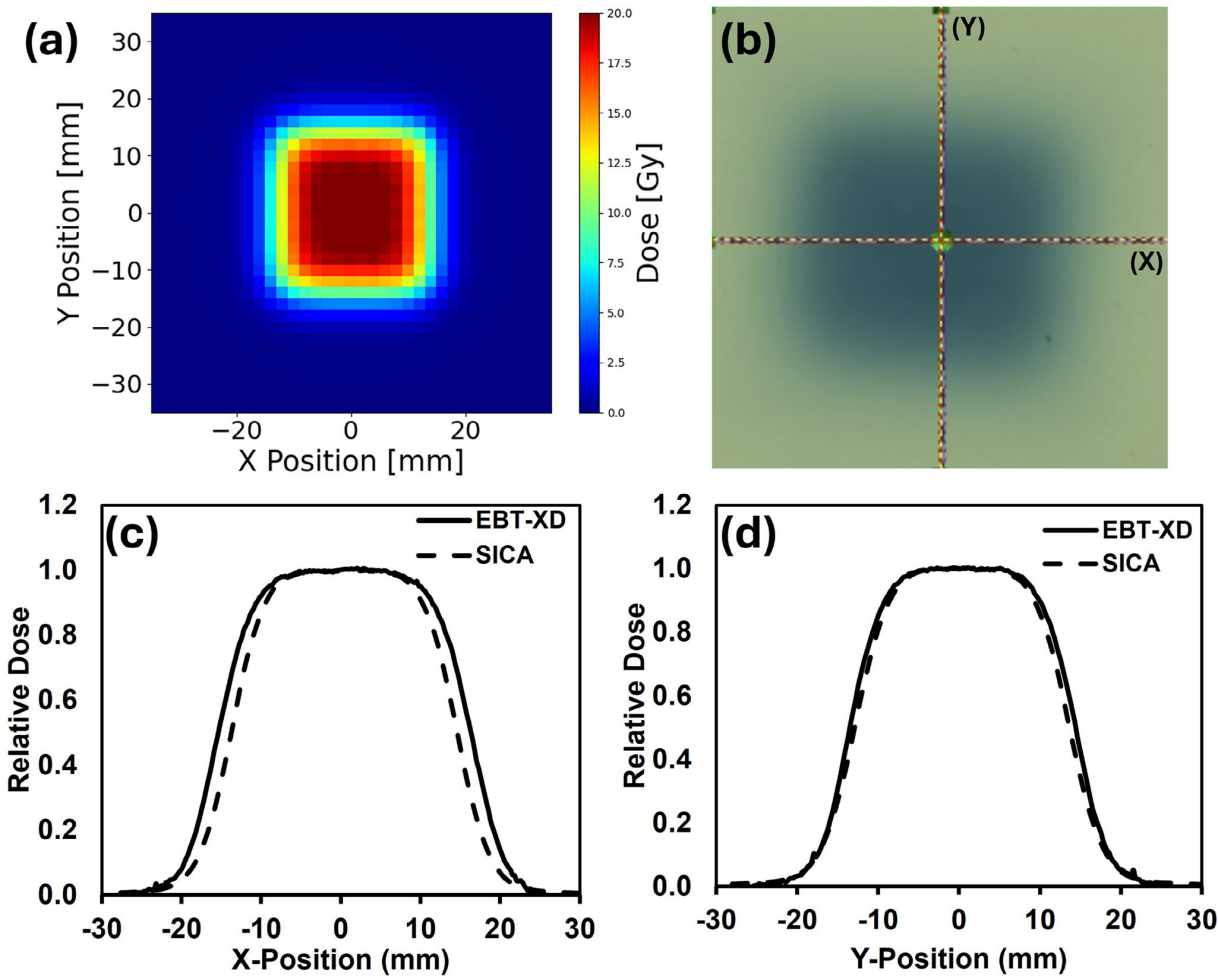
The film was used to compare the SICA measurement for the 2D and line dose profile. (a) SICA 2D dose distribution, (b) EBT‐XD film 2D dose, (c) X‐profile (SICA vs. EBT‐XD), (d) Y‐profile (SICA vs. EBT‐XD).

Figure 3 presents a comprehensive comparison of 2D dose distributions and profiles obtained from EBT‐XD film measurements and the SICA for the same irradiation field in the one‐shot setup. The 2D dose distribution from the SICA and EBT‐XD film measurement were demonstrated (Figure 3a,b). Beam profile measurements were conducted at the nozzle, and the film was affixed directly to the distal face of the SICA detector to ensure spatial correspondence. These profiles were acquired using a nozzle beam current of 215 nA. For SICA, the 80%–20% penumbra of the *X*‐ and *Y*‐profile were measured at 5.85 and 5.85 mm, and for the film, the values were 5.85 and 5.46 mm. Analyses revealed excellent agreement in the central plateau region between both detection methods, while minor discrepancies were observed in the penumbra regions (Figure 3c,d). These variations are potentially due to the asymmetric cross‐beam profile of the pencil beam, which is more accurately captured by the film.

### 3.3 Quantification of 2‐D dose rate

**FIGURE 4:**
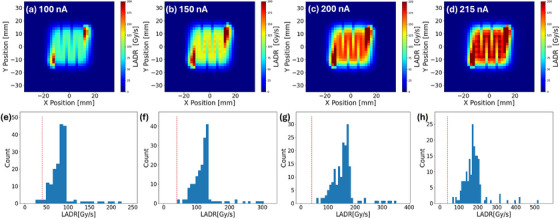
The 2D dose rate distribution of SICA using the local average dose rate method. (a)–(d) show the dose rate distributions for beam currents of 100, 150, 200, and 215 nA, respectively. (e)–(f) show the corresponding LADR dose rate histograms. LADR above 40 Gy/s is indicated by the vertical dashed lines. Histograms only included voxels exceeding a 10% dose threshold.

The LADR method calculates the dose rate for individual voxels by determining the ratio of the delivered dose to the duration of significant dose accumulation, which occurs when the pencil beam is near the voxel under consideration. Figure 4 comprehensively presents our LADR measurements of SICA. Panels (a) to (d) display the 2D dose rate distributions for each beam current. Although the overall dose distribution appears uniform, these maps show substantial variations in local dose rates throughout the field, characterized by a distinctive zig‐zag pattern. Notably, the spatial patterns of dose rate variations remain consistent across all beam currents, suggesting they are intrinsic to the delivery system, field geometry, and scanning pattern, rather than current‐dependent. Additionally, panels (e) to (h) present histograms of the LADR dose rates for each beam current. As shown in Figure 4e–h, the LADR exceeded 40 Gy/s for all tested currents, indicating that the radiation delivery operates within the FLASH regime. As the beam current increases, we observe the histogram shifted to a higher dose rate end. These results underscored the complex nature of dose rate distributions in proton PBS therapy and emphasized the importance of considering the time structure and local dose accumulation from spots.

**FIGURE 5:**
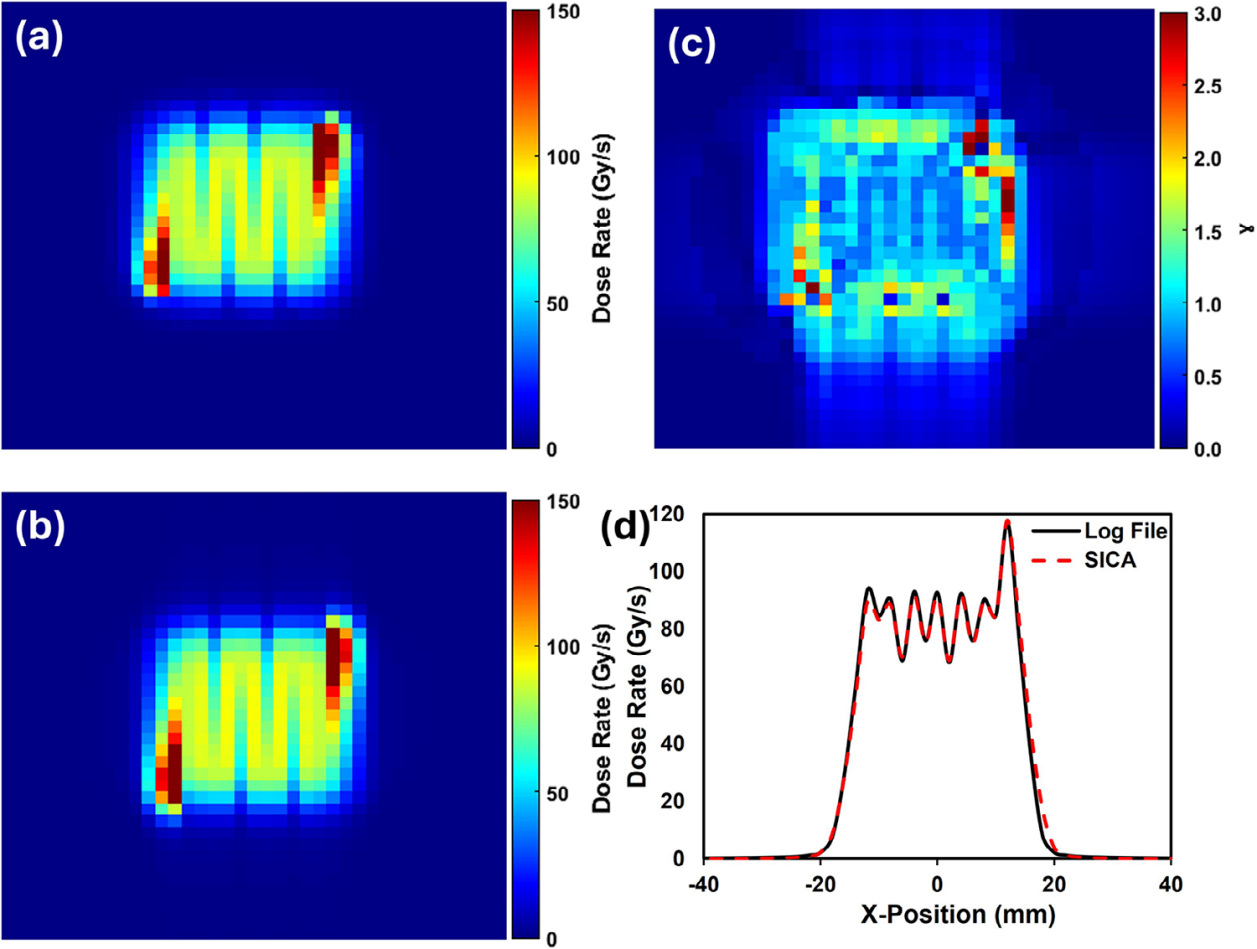
Dose rate reconstruction of the (a) SICA and (b) log file measurement for 100 nA delivery. (c) the gamma passing rate distribution, and (d) the line profile (red dotted line in panel a) comparison between the log file and measurement.

Subsequently, the dose rate reconstruction analysis was conducted for both the treatment log file and the SICA measurement at a beam current of 100 nA (Figure 5). A comparison of these two datasets revealed notable similarities in the overall dose rate patterns, suggesting good agreement between the planned and delivered treatments. To quantify the level of agreement between the planned dose rates (derived from the log file) and the measured dose rates (obtained from SICA), a gamma analysis was performed with a 10% dose threshold as the cutoff. The resulting gamma passing rate (94.9%) distribution demonstrated high concordance between the two datasets across the majority of the treatment field. Areas of lower agreement, indicated by lower gamma passing rates, were primarily observed at the field edges, which is consistent with the challenges typically associated with accurate dose delivery in high‐gradient regions.

This one‐dimensional analysis provided detailed insights into the spatial variations of dose rate agreement. The line profiles exhibited close alignment in the central regions of the field, with little discrepancy noted towards the field boundaries. These discrepancies may be attributed to factors such as beam penumbra effects, positioning uncertainties, or limitations in the detection system's spatial resolution. The collective analysis of the dose rate reconstructions, gamma passing rate distribution, and line profile comparisons offers a comprehensive assessment of the treatment delivery accuracy. These results underscore the importance of rigorous QA procedures in advanced radiotherapy techniques, particularly when employing high dose rates that approach or exceed the FLASH threshold.

## 4 DISCUSSION

In this work, we present an effective routine calibration and QA procedure for proton FLASH delivery designed to ensure high‐quality dosimetry performance in both preclinical and clinical settings. The accuracy and effectiveness of the proposed procedure have been verified through third‐party credentialing, further demonstrating its reliability and robustness. This verification process underscores the potential of the procedure to be adopted widely, facilitating consistent and accurate dosimetry, that is a current technology gap but essential for the success of future proton FLASH clinical trials.

Absolute dosimetry in proton FLASH delivery poses significant challenges, primarily due to the need for high spatiotemporal resolution detectors. These detectors are crucial for capturing the rapid dose delivery characteristic of FLASH‐RT. However, the availability of such detectors is currently limited, posing a barrier to achieving the desired precision in dosimetric measurements and QA.^31^ The scarcity of high‐resolution detectors highlights the need for ongoing development and optimization of dosimetry tools to meet the rigorous demands of proton FLASH delivery.

Additionally, the SICA system, while a valuable tool for monitoring, is not yet an active component integrated into the delivery system. Its current function is limited to providing monitoring capabilities without the ability to control the delivery process. This limitation restricts its utility in real‐time dosimetric adjustments during treatment, emphasizing the need for further development to integrate active control features. Such advancements would enhance the capability of SICA to not only monitor but also dynamically adjust the dosimetry, ensuring even greater accuracy and safety in proton FLASH delivery. While our workflow incorporates SICA technology for enhanced precision, we recognize the importance of providing approaches for diverse institutional settings. For institutions without access to SICA technology, we propose two complementary strategies: (1) implementation of multi‐detector validation protocols where measurements from different detector types (e.g., radiochromic film and small‐volume ionization chambers) are cross‐verified to ensure consistency in the absence of SICA's precision,^17^ and (2) establishment of inter‐institutional calibration networks where centers can periodically benchmark their systems against reference measurements from SICA‐equipped facilities.

Overall, the proposed QA procedure for proton FLASH delivery demonstrates significant potential in enhancing dosimetric accuracy and reliability. However, the challenges associated with absolute dosimetry and the current limitations of the SICA system indicate areas where further technological advancements are necessary. Continued innovation in detector technology and the integration of active control mechanisms into monitoring systems like SICA will be essential to fully realize the benefits of proton FLASH therapy in clinical practice.

## 5 CONCLUSION

This technical note successfully establishes a robust and effective calibration and QA procedure for proton FLASH delivery, ensuring high passing rates and validation by third‐party credentialing bodies. The findings demonstrate that the dose can be delivered with remarkable accuracy, with the agreement between planned and delivered doses consistently within 1%. This high level of precision provides a solid reference in dosimetry, which is crucial for the advancement of future clinical trial applications. These results demonstrated the reliability and efficacy of the proposed methods, paving the way for their broader adoption in clinical settings and contributing to the enhancement of proton FLASH‐RT preclinical and clinical applications.

## AUTHOR CONTRIBUTIONS

Chih‐Chiang Chang and Minglei Kang contributed to initial drafting; Minglei Kang developed the conceptualization; Balaji Selvaraj and Chih‐Chiang Chang conducted data processing; Haibo Lin and Minglei Kang performed data curation; all authors participated in writing and editing the manuscript.

## CONFLICT OF INTEREST STATEMENT

The authors declare no conflicts of interest.
